# (2-Ethyl-2-oxazoline-κ*N*)bis(*N*-ethyl-*N*-phenyl­dithio­carbamato-κ^2^ *S*,*S*′)cadmium

**DOI:** 10.1107/S1600536812038433

**Published:** 2012-09-29

**Authors:** Damian C. Onwudiwe, Christien A. Strydom, Eric C. Hosten

**Affiliations:** a Chemical Resource Beneficiation, North-West University, Private Bag X6001, Potchefstroom 2520, South Africa; b Department of Chemistry, Nelson Mandela Metropolitan University, PO Box 77000, Port Elizabeth 6031, South Africa

## Abstract

In the title compound, [Cd(C_9_H_10_NS_2_)_2_(C_5_H_9_NO)], the Cd^II^ atom is five-coordinated in a distorted square-pyramidal geometry by four S atoms from two chelating *N*-ethyl-*N*-phenyl dithio­carbamate ligands and one N atom from a 2-ethyl-2-oxazoline ligand. Inter­molecular C—H⋯π inter­actions are observed in the crystal structure.

## Related literature

For background to and applications of dithio­carbamates, see: Green *et al.* (2004 ▶); Pickett & O’Brien (2001 ▶); Tiekink (2003 ▶); Valarmathi *et al.* (2011 ▶). For the synthesis of the parent dithio­carbamate, see: Onwudiwe & Ajibade (2010 ▶). For information regarding dithio­carbanate adducts, see: Green & O’Brien (1997 ▶); Ivanov *et al.* (2007 ▶); Onwudiwe *et al.* (2011 ▶). For the synthesis and structures of dithio­carbamates incorporating oxazoline mol­ecules, see: Decken *et al.* (2006 ▶); Gossage & Jenkins (2008 ▶).
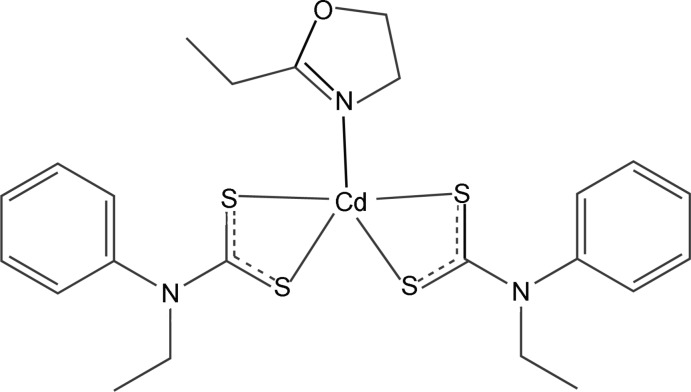


## Experimental

### 

#### Crystal data

[Cd(C_9_H_10_NS_2_)_2_(C_5_H_9_NO)]
*M* *_r_* = 604.18
Triclinic, 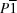

*a* = 10.3119 (2) Å
*b* = 11.4395 (2) Å
*c* = 12.2432 (3) Å
α = 84.756 (1)°
β = 77.395 (1)°
γ = 70.290 (1)°
*V* = 1326.61 (5) Å^3^
*Z* = 2
Mo *K*α radiation
μ = 1.16 mm^−1^
*T* = 200 K
0.37 × 0.23 × 0.18 mm

#### Data collection

Bruker APEXII CCD diffractometer
Absorption correction: multi-scan (*SADABS*; Bruker, 2001 ▶) *T*_min_ = 0.75, *T*_max_ = 0.82
23482 measured reflections
6626 independent reflections
5934 reflections with *I* > 2σ(*I*)
*R*_int_ = 0.017

#### Refinement

*R*[*F* ^2^ > 2σ(*F* ^2^)] = 0.022
*wR*(*F* ^2^) = 0.054
*S* = 1.06
6626 reflections
292 parameters
H-atom parameters constrained
Δρ_max_ = 0.67 e Å^−3^
Δρ_min_ = −0.41 e Å^−3^

### 

Data collection: *APEX2* (Bruker, 2007 ▶); cell refinement: *SAINT* (Bruker, 2007 ▶); data reduction: *SAINT*; program(s) used to solve structure: *SHELXS97* (Sheldrick, 2008 ▶); program(s) used to refine structure: *SHELXL97* (Sheldrick, 2008 ▶); molecular graphics: *ORTEP-3* (Farrugia, 1997 ▶); software used to prepare material for publication: *PLATON* (Spek, 2009 ▶) and *publCIF* (Westrip, 2010 ▶).

## Supplementary Material

Crystal structure: contains datablock(s) global, I. DOI: 10.1107/S1600536812038433/hy2583sup1.cif

Structure factors: contains datablock(s) I. DOI: 10.1107/S1600536812038433/hy2583Isup2.hkl

Supplementary material file. DOI: 10.1107/S1600536812038433/hy2583Isup3.cdx

Additional supplementary materials:  crystallographic information; 3D view; checkCIF report

## Figures and Tables

**Table 1:** Hydrogen-bond geometry (Å, °) *Cg*1 and *Cg*2 are the centroids of the C11–C16 and C21–C26 rings, respectively.

*D*—H⋯*A*	*D*—H	H⋯*A*	*D*⋯*A*	*D*—H⋯*A*
C26—H26⋯*Cg*1^i^	0.95	2.61	3.558 (2)	177
C32—H32*A*⋯*Cg*1^ii^	0.99	2.72	3.511 (2)	137
C13—H13⋯*Cg*2^iii^	0.95	2.61	3.510 (2)	157

Symmetry codes: (i) 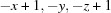; (ii) 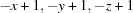; (iii) 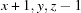.

**Fig. 1.**
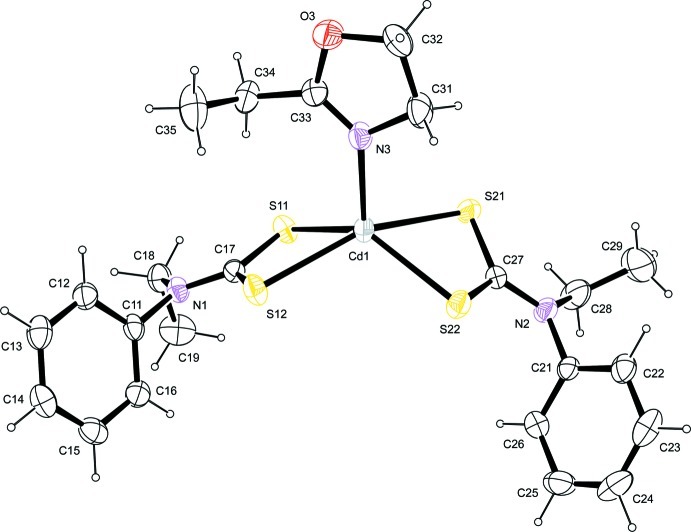
The molecular structure of the title compound. Displacement ellipsoids are drawn at the 50% probability level.

**Fig. 2.**
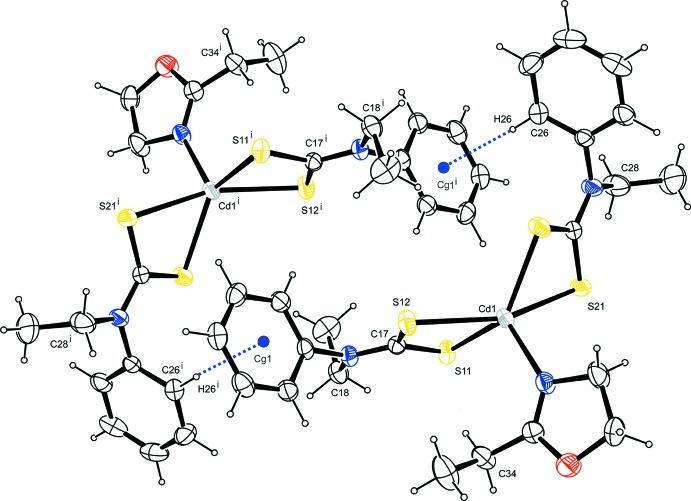
Selected intermolecular C—H···π contacts (blue dashed lines). [Cg1 is the centroid of the C11–C16 ring. Symmetry code: (i) -x+1, -y, -z+1.]

**Fig. 3.**
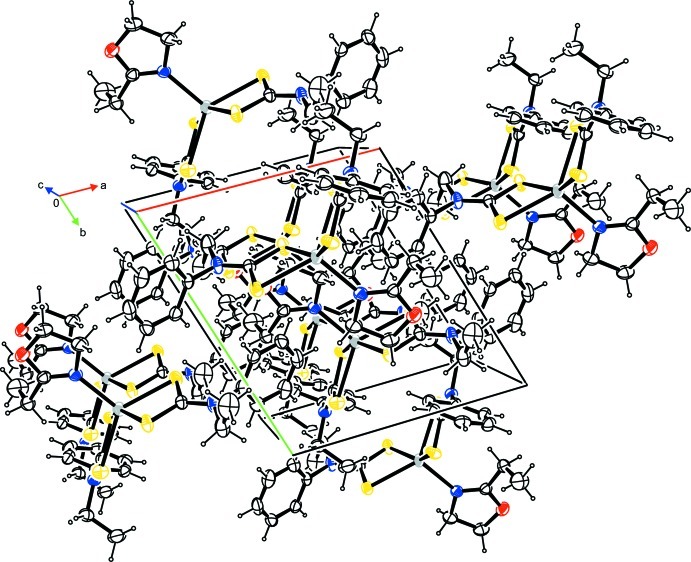
Crystal packing of the title compound, viewed along the c-axis.

## supplementary crystallographic information

### Comment

One of the attractive features of group 12 dithiocarbamate chemistry is the extensive structural motifs which they display, ranging from monomeric, dimeric, tetrameric, linear polymeric and layered structures (Tiekink, 2003). These compounds tend to reversibly add organic N-, O-, S- and P-donor bases to give heteroligand complexes generally called adducts (Ivanov *et al.*, 2007). Such adducts are of practical interest as they display a wide range of applications (Green *et al.*, 2004; Pickett & O'Brien, 2001; Valarmathi *et al.*, 2011). The molecules are usually highly volatile and are used in improved synthesis of nanoparticulate chalcogenide semiconductors, with good luminescent properties (Green and O'Brien, 1997). As part of our interest in the studies of N-donor adducts of group 12 dithiocarbamates (Onwudiwe *et al.*, 2011), the structure analysis of the title compound was undertaken.

The Cd^II^ atom in the title compound is square-pyramidal five coordinate with four S atoms from two *N*-ethyl-*N*-phenyl dithiocarbamate ligands and one N atom from a 2-ethyl-2-oxazoline ligand (Fig. 1). The two dithiocarbamates are at an obtuse angle of 130.6 ° to each other and form an angle of 89.8 ° and 85.6 ° with the oxazoline ligand. The Cd atom is 0.7877 (1) Å above the plane formed by the four S atoms. The Cd—S bond lengths vary from 2.5615 (5) to 2.7154 (4) Å while the Cd—N bond length is 2.2564 (14) Å. None of the ethyl groups shows any signifuicant disorder. The dithiocarbamate ethyl groups have intramolecular interactions with the S atoms C18—H18A···S11 and C28—H28A···S21, with contact distances of 2.60 and 2.56 Å respectively. Adjacent molecules are linked by C—H···π interactions (Table 1, Fig. 2). Packing of the title compound is shown in Fig. 3.

### Experimental

(*N*-Ethyl-*N*-phenyl dithiocarbamate)cadmium (2 mmol, 1.01 g) was suspended in 75 ml of warm dichloromethane (Onwudiwe & Ajibade, 2010). 2-Ethyl-2-oxazoline was dropwise added to the stirring warm mixture. The clear solution obtained after the addition of oxazoline was stirred for 10 h. The colourless solution obtained was filtered and the solvent was removed. The resulting crude product was redissolved in boiling acetone (Decken *et al.*, 2006; Gossage & Jenkins, 2008). After a few days, single crystals suitable for X-ray structure analysis were obtained (m.p. 288–290 °C).

### Refinement

H atoms were placed in calculated positions and refined as riding atoms, with C—H = 0.95 (CH), 0.99 (CH_2_) and 0.98 (CH_3_) Å and with *U*_iso_(H) = 1.2(1.5 for methyl)*U*_eq_(C).

### Crystal data

**Table:**

[Cd(C_9_H_10_NS_2_)_2_(C_5_H_9_NO)]	*Z* = 2
*M**_r_* = 604.18	*F*(000) = 616
Triclinic, *P*1	*D*_x_ = 1.513 Mg m^−^^3^
Hall symbol: -P 1	Melting point: 562.15 K
*a* = 10.3119 (2) Å	Mo *K*α radiation, λ = 0.71073 Å
*b* = 11.4395 (2) Å	Cell parameters from 192 reflections
*c* = 12.2432 (3) Å	θ = 1.7–25.5°
α = 84.756 (1)°	µ = 1.16 mm^−^^1^
β = 77.395 (1)°	*T* = 200 K
γ = 70.290 (1)°	Block, colourless
*V* = 1326.61 (5) Å^3^	0.37 × 0.23 × 0.18 mm

### Data collection

**Table:**

Bruker APEXII CCD diffractometer	6626 independent reflections
Radiation source: sealed tube	5934 reflections with *I* > 2σ(*I*)
Graphite monochromator	*R*_int_ = 0.017
φ and ω scans	θ_max_ = 28.4°, θ_min_ = 2.1°
Absorption correction: multi-scan (*SADABS*; Bruker, 2001)	*h* = −13→13
*T*_min_ = 0.75, *T*_max_ = 0.82	*k* = −15→15
23482 measured reflections	*l* = −16→16

### Refinement

**Table:**

Refinement on *F*^2^	Primary atom site location: structure-invariant direct methods
Least-squares matrix: full	Secondary atom site location: difference Fourier map
*R*[*F*^2^ > 2σ(*F*^2^)] = 0.022	Hydrogen site location: inferred from neighbouring sites
*wR*(*F*^2^) = 0.054	H-atom parameters constrained
*S* = 1.06	*w* = 1/[σ^2^(*F*_o_^2^) + (0.0233*P*)^2^ + 0.6233*P*] where *P* = (*F*_o_^2^ + 2*F*_c_^2^)/3
6626 reflections	(Δ/σ)_max_ = 0.001
292 parameters	Δρ_max_ = 0.67 e Å^−^^3^
0 restraints	Δρ_min_ = −0.41 e Å^−^^3^

### Special details

**Table:**

Geometry. All e.s.d.'s (except the e.s.d. in the dihedral angle between two l.s. planes) are estimated using the full covariance matrix. The cell e.s.d.'s are taken into account individually in the estimation of e.s.d.'s in distances, angles and torsion angles; correlations between e.s.d.'s in cell parameters are only used when they are defined by crystal symmetry. An approximate (isotropic) treatment of cell e.s.d.'s is used for estimating e.s.d.'s involving l.s. planes.
Refinement. Refinement of *F*^2^ against ALL reflections. The weighted *R*-factor *wR* and goodness of fit *S* are based on *F*^2^, conventional *R*-factors *R* are based on *F*, with *F* set to zero for negative *F*^2^. The threshold expression of *F*^2^ > σ(*F*^2^) is used only for calculating *R*-factors(gt) *etc*. and is not relevant to the choice of reflections for refinement. *R*-factors based on *F*^2^ are statistically about twice as large as those based on *F*, and *R*- factors based on ALL data will be even larger.

### Fractional atomic coordinates and isotropic or equivalent isotropic displacement parameters (Å^2^)

**Table:**

	*x*	*y*	*z*	*U*_iso_*/*U*_eq_	
Cd1	0.497101 (12)	0.297575 (11)	0.710351 (10)	0.02896 (4)	
S11	0.70543 (5)	0.08672 (4)	0.66862 (3)	0.03257 (9)	
S12	0.55149 (5)	0.24235 (4)	0.49921 (3)	0.03339 (9)	
S21	0.42738 (4)	0.25089 (4)	0.93305 (3)	0.03282 (9)	
S22	0.23016 (4)	0.35727 (4)	0.77674 (3)	0.03088 (9)	
O3	0.70297 (14)	0.58511 (12)	0.68191 (11)	0.0403 (3)	
N1	0.75835 (14)	0.02943 (12)	0.45349 (11)	0.0269 (3)	
N2	0.16026 (15)	0.25674 (15)	0.97431 (11)	0.0316 (3)	
N3	0.56806 (15)	0.46441 (12)	0.70659 (11)	0.0293 (3)	
C11	0.73383 (17)	0.04933 (14)	0.34024 (13)	0.0263 (3)	
C12	0.81004 (19)	0.11050 (16)	0.26380 (14)	0.0325 (4)	
H12	0.8768	0.1391	0.2858	0.039*	
C13	0.7883 (2)	0.12968 (16)	0.15508 (15)	0.0369 (4)	
H13	0.8394	0.1725	0.1024	0.044*	
C14	0.6923 (2)	0.08674 (16)	0.12285 (15)	0.0357 (4)	
H14	0.6777	0.1001	0.0481	0.043*	
C15	0.61753 (19)	0.02427 (15)	0.19940 (15)	0.0341 (4)	
H15	0.552	−0.0056	0.177	0.041*	
C16	0.63808 (18)	0.00527 (15)	0.30856 (14)	0.0303 (3)	
H16	0.587	−0.0376	0.3612	0.036*	
C17	0.67929 (17)	0.11157 (14)	0.53306 (13)	0.0256 (3)	
C18	0.86741 (19)	−0.08783 (16)	0.47616 (15)	0.0336 (4)	
H18A	0.9061	−0.0765	0.5402	0.04*	
H18B	0.9452	−0.1084	0.41	0.04*	
C19	0.8089 (2)	−0.19382 (18)	0.5024 (2)	0.0489 (5)	
H19A	0.7793	−0.2108	0.4364	0.073*	
H19B	0.7279	−0.1714	0.5649	0.073*	
H19C	0.8815	−0.2681	0.5231	0.073*	
C21	0.02419 (16)	0.28275 (15)	0.94601 (13)	0.0262 (3)	
C22	−0.08658 (19)	0.38602 (16)	0.98745 (15)	0.0345 (4)	
H22	−0.0734	0.4425	1.0328	0.041*	
C23	−0.2169 (2)	0.40647 (19)	0.96237 (17)	0.0430 (5)	
H23	−0.2931	0.4784	0.9889	0.052*	
C24	−0.2363 (2)	0.3226 (2)	0.89888 (17)	0.0453 (5)	
H24	−0.3266	0.3357	0.8835	0.054*	
C25	−0.1251 (2)	0.2200 (2)	0.85763 (16)	0.0434 (5)	
H25	−0.1387	0.1631	0.813	0.052*	
C26	0.00623 (19)	0.19937 (17)	0.88082 (14)	0.0335 (4)	
H26	0.0831	0.1287	0.8523	0.04*	
C27	0.26380 (16)	0.28540 (15)	0.90230 (13)	0.0258 (3)	
C28	0.1801 (2)	0.1855 (2)	1.08128 (16)	0.0456 (5)	
H28A	0.2819	0.1458	1.0796	0.055*	
H28B	0.1375	0.1189	1.0878	0.055*	
C29	0.1152 (3)	0.2663 (3)	1.18189 (18)	0.0654 (7)	
H29A	0.1547	0.3341	1.1747	0.098*	
H29B	0.1352	0.2166	1.2495	0.098*	
H29C	0.0132	0.3009	1.1871	0.098*	
C31	0.4824 (2)	0.57458 (17)	0.77370 (17)	0.0422 (4)	
H31A	0.4604	0.5516	0.8539	0.051*	
H31B	0.3933	0.6169	0.7476	0.051*	
C32	0.5742 (2)	0.65695 (17)	0.75524 (16)	0.0389 (4)	
H32A	0.5287	0.737	0.7191	0.047*	
H32B	0.5935	0.6736	0.827	0.047*	
C33	0.68437 (18)	0.47990 (15)	0.66044 (14)	0.0292 (3)	
C34	0.80295 (19)	0.39458 (17)	0.58279 (15)	0.0366 (4)	
H34A	0.7899	0.3124	0.586	0.044*	
H34B	0.8922	0.3833	0.6067	0.044*	
C35	0.8113 (3)	0.4459 (2)	0.46306 (18)	0.0601 (6)	
H35A	0.8927	0.39	0.4141	0.09*	
H35B	0.8214	0.5283	0.4603	0.09*	
H35C	0.7253	0.4523	0.4378	0.09*	

### Atomic displacement parameters (Å^2^)

**Table:**

	*U*^11^	*U*^22^	*U*^33^	*U*^12^	*U*^13^	*U*^23^
Cd1	0.02741 (7)	0.03168 (7)	0.02647 (6)	−0.01143 (5)	0.00288 (4)	−0.00717 (4)
S11	0.0394 (2)	0.0327 (2)	0.02396 (19)	−0.00906 (18)	−0.00617 (17)	−0.00305 (15)
S12	0.0342 (2)	0.0327 (2)	0.02624 (19)	−0.00064 (17)	−0.00540 (17)	−0.00694 (16)
S21	0.0250 (2)	0.0492 (2)	0.0274 (2)	−0.01531 (18)	−0.00557 (16)	−0.00294 (17)
S22	0.0253 (2)	0.0397 (2)	0.02583 (19)	−0.01067 (17)	−0.00322 (15)	0.00416 (16)
O3	0.0424 (7)	0.0316 (6)	0.0481 (8)	−0.0174 (6)	−0.0010 (6)	−0.0048 (5)
N1	0.0295 (7)	0.0242 (6)	0.0247 (6)	−0.0070 (5)	−0.0023 (5)	−0.0033 (5)
N2	0.0252 (7)	0.0490 (9)	0.0226 (6)	−0.0160 (6)	−0.0039 (5)	0.0016 (6)
N3	0.0298 (7)	0.0258 (6)	0.0285 (7)	−0.0071 (6)	0.0002 (6)	−0.0040 (5)
C11	0.0304 (8)	0.0216 (7)	0.0232 (7)	−0.0062 (6)	0.0009 (6)	−0.0054 (6)
C12	0.0361 (9)	0.0307 (8)	0.0315 (8)	−0.0155 (7)	0.0008 (7)	−0.0044 (7)
C13	0.0431 (10)	0.0317 (8)	0.0304 (9)	−0.0121 (8)	0.0024 (7)	0.0024 (7)
C14	0.0423 (10)	0.0307 (8)	0.0276 (8)	−0.0035 (7)	−0.0060 (7)	−0.0036 (7)
C15	0.0368 (9)	0.0277 (8)	0.0374 (9)	−0.0069 (7)	−0.0096 (7)	−0.0083 (7)
C16	0.0318 (9)	0.0257 (7)	0.0322 (8)	−0.0109 (7)	−0.0006 (7)	−0.0035 (6)
C17	0.0271 (8)	0.0268 (7)	0.0239 (7)	−0.0123 (6)	−0.0006 (6)	−0.0034 (6)
C18	0.0313 (9)	0.0306 (8)	0.0329 (9)	−0.0040 (7)	−0.0024 (7)	−0.0046 (7)
C19	0.0559 (13)	0.0295 (9)	0.0625 (13)	−0.0110 (9)	−0.0224 (11)	0.0062 (9)
C21	0.0241 (8)	0.0348 (8)	0.0213 (7)	−0.0136 (6)	−0.0017 (6)	0.0006 (6)
C22	0.0343 (9)	0.0313 (8)	0.0367 (9)	−0.0160 (7)	0.0046 (7)	−0.0029 (7)
C23	0.0279 (9)	0.0395 (10)	0.0506 (11)	−0.0070 (8)	0.0029 (8)	0.0120 (8)
C24	0.0294 (9)	0.0681 (14)	0.0429 (11)	−0.0235 (9)	−0.0141 (8)	0.0231 (10)
C25	0.0487 (12)	0.0616 (13)	0.0332 (9)	−0.0317 (10)	−0.0142 (8)	0.0010 (9)
C26	0.0340 (9)	0.0377 (9)	0.0282 (8)	−0.0115 (7)	−0.0034 (7)	−0.0058 (7)
C27	0.0242 (8)	0.0301 (8)	0.0231 (7)	−0.0095 (6)	−0.0014 (6)	−0.0062 (6)
C28	0.0390 (11)	0.0690 (14)	0.0330 (10)	−0.0249 (10)	−0.0093 (8)	0.0121 (9)
C29	0.0788 (18)	0.099 (2)	0.0295 (10)	−0.0434 (16)	−0.0114 (11)	0.0008 (11)
C31	0.0410 (10)	0.0313 (9)	0.0446 (11)	−0.0061 (8)	0.0069 (8)	−0.0124 (8)
C32	0.0496 (11)	0.0295 (8)	0.0354 (9)	−0.0090 (8)	−0.0069 (8)	−0.0077 (7)
C33	0.0328 (9)	0.0259 (7)	0.0281 (8)	−0.0102 (7)	−0.0048 (7)	0.0030 (6)
C34	0.0323 (9)	0.0337 (9)	0.0373 (9)	−0.0091 (7)	0.0042 (7)	−0.0010 (7)
C35	0.0701 (16)	0.0582 (14)	0.0361 (11)	−0.0131 (12)	0.0092 (10)	−0.0006 (10)

### Geometric parameters (Å, º)

**Table:**

Cd1—N3	2.2563 (14)	C19—H19A	0.98
Cd1—S22	2.5615 (4)	C19—H19B	0.98
Cd1—S12	2.6121 (4)	C19—H19C	0.98
Cd1—S11	2.6354 (5)	C21—C26	1.380 (2)
Cd1—S21	2.7154 (4)	C21—C22	1.380 (2)
S11—C17	1.7221 (16)	C22—C23	1.382 (3)
S12—C17	1.7152 (17)	C22—H22	0.95
S21—C27	1.7157 (16)	C23—C24	1.378 (3)
S22—C27	1.7230 (16)	C23—H23	0.95
O3—C33	1.338 (2)	C24—C25	1.377 (3)
O3—C32	1.457 (2)	C24—H24	0.95
N1—C17	1.342 (2)	C25—C26	1.382 (3)
N1—C11	1.447 (2)	C25—H25	0.95
N1—C18	1.481 (2)	C26—H26	0.95
N2—C27	1.341 (2)	C28—C29	1.500 (3)
N2—C21	1.445 (2)	C28—H28A	0.99
N2—C28	1.493 (2)	C28—H28B	0.99
N3—C33	1.272 (2)	C29—H29A	0.98
N3—C31	1.472 (2)	C29—H29B	0.98
C11—C12	1.383 (2)	C29—H29C	0.98
C11—C16	1.385 (2)	C31—C32	1.518 (3)
C12—C13	1.384 (3)	C31—H31A	0.99
C12—H12	0.95	C31—H31B	0.99
C13—C14	1.382 (3)	C32—H32A	0.99
C13—H13	0.95	C32—H32B	0.99
C14—C15	1.385 (3)	C33—C34	1.486 (2)
C14—H14	0.95	C34—C35	1.524 (3)
C15—C16	1.385 (2)	C34—H34A	0.99
C15—H15	0.95	C34—H34B	0.99
C16—H16	0.95	C35—H35A	0.98
C18—C19	1.508 (3)	C35—H35B	0.98
C18—H18A	0.99	C35—H35C	0.98
C18—H18B	0.99		
			
N3—Cd1—S22	110.83 (4)	C22—C21—N2	120.50 (15)
N3—Cd1—S12	103.12 (4)	C21—C22—C23	119.38 (17)
S22—Cd1—S12	106.646 (14)	C21—C22—H22	120.3
N3—Cd1—S11	113.83 (4)	C23—C22—H22	120.3
S22—Cd1—S11	134.900 (15)	C24—C23—C22	120.08 (18)
S12—Cd1—S11	69.059 (13)	C24—C23—H23	120.0
N3—Cd1—S21	102.68 (4)	C22—C23—H23	120.0
S22—Cd1—S21	68.706 (13)	C25—C24—C23	120.13 (17)
S12—Cd1—S21	153.616 (15)	C25—C24—H24	119.9
S11—Cd1—S21	95.366 (14)	C23—C24—H24	119.9
C17—S11—Cd1	85.11 (6)	C24—C25—C26	120.36 (18)
C17—S12—Cd1	85.98 (5)	C24—C25—H25	119.8
C27—S21—Cd1	82.05 (5)	C26—C25—H25	119.8
C27—S22—Cd1	86.74 (5)	C21—C26—C25	119.11 (17)
C33—O3—C32	106.82 (13)	C21—C26—H26	120.4
C17—N1—C11	120.28 (13)	C25—C26—H26	120.4
C17—N1—C18	123.19 (14)	N2—C27—S21	121.16 (12)
C11—N1—C18	116.43 (13)	N2—C27—S22	118.67 (12)
C27—N2—C21	120.66 (13)	S21—C27—S22	120.16 (9)
C27—N2—C28	123.45 (14)	N2—C28—C29	112.39 (19)
C21—N2—C28	115.58 (13)	N2—C28—H28A	109.1
C33—N3—C31	107.76 (14)	C29—C28—H28A	109.1
C33—N3—Cd1	130.29 (11)	N2—C28—H28B	109.1
C31—N3—Cd1	121.64 (11)	C29—C28—H28B	109.1
C12—C11—C16	120.73 (15)	H28A—C28—H28B	107.9
C12—C11—N1	118.80 (15)	C28—C29—H29A	109.5
C16—C11—N1	120.45 (14)	C28—C29—H29B	109.5
C11—C12—C13	119.47 (16)	H29A—C29—H29B	109.5
C11—C12—H12	120.3	C28—C29—H29C	109.5
C13—C12—H12	120.3	H29A—C29—H29C	109.5
C14—C13—C12	120.22 (16)	H29B—C29—H29C	109.5
C14—C13—H13	119.9	N3—C31—C32	104.14 (15)
C12—C13—H13	119.9	N3—C31—H31A	110.9
C13—C14—C15	120.05 (16)	C32—C31—H31A	110.9
C13—C14—H14	120.0	N3—C31—H31B	110.9
C15—C14—H14	120.0	C32—C31—H31B	110.9
C16—C15—C14	120.12 (16)	H31A—C31—H31B	108.9
C16—C15—H15	119.9	O3—C32—C31	103.97 (13)
C14—C15—H15	119.9	O3—C32—H32A	111.0
C15—C16—C11	119.40 (15)	C31—C32—H32A	111.0
C15—C16—H16	120.3	O3—C32—H32B	111.0
C11—C16—H16	120.3	C31—C32—H32B	111.0
N1—C17—S12	119.65 (12)	H32A—C32—H32B	109.0
N1—C17—S11	120.50 (12)	N3—C33—O3	117.29 (15)
S12—C17—S11	119.85 (9)	N3—C33—C34	127.25 (15)
N1—C18—C19	111.61 (15)	O3—C33—C34	115.45 (15)
N1—C18—H18A	109.3	C33—C34—C35	110.98 (16)
C19—C18—H18A	109.3	C33—C34—H34A	109.4
N1—C18—H18B	109.3	C35—C34—H34A	109.4
C19—C18—H18B	109.3	C33—C34—H34B	109.4
H18A—C18—H18B	108.0	C35—C34—H34B	109.4
C18—C19—H19A	109.5	H34A—C34—H34B	108.0
C18—C19—H19B	109.5	C34—C35—H35A	109.5
H19A—C19—H19B	109.5	C34—C35—H35B	109.5
C18—C19—H19C	109.5	H35A—C35—H35B	109.5
H19A—C19—H19C	109.5	C34—C35—H35C	109.5
H19B—C19—H19C	109.5	H35A—C35—H35C	109.5
C26—C21—C22	120.92 (16)	H35B—C35—H35C	109.5
C26—C21—N2	118.52 (15)		
			
N3—Cd1—S11—C17	−95.97 (6)	Cd1—S12—C17—N1	179.40 (12)
S22—Cd1—S11—C17	92.53 (5)	Cd1—S12—C17—S11	−0.71 (9)
S12—Cd1—S11—C17	−0.43 (5)	Cd1—S11—C17—N1	−179.41 (13)
S21—Cd1—S11—C17	157.62 (5)	Cd1—S11—C17—S12	0.70 (8)
N3—Cd1—S12—C17	111.22 (6)	C17—N1—C18—C19	91.8 (2)
S22—Cd1—S12—C17	−131.98 (5)	C11—N1—C18—C19	−84.61 (19)
S11—Cd1—S12—C17	0.43 (5)	C27—N2—C21—C26	82.5 (2)
S21—Cd1—S12—C17	−56.47 (6)	C28—N2—C21—C26	−91.38 (19)
N3—Cd1—S21—C27	116.69 (6)	C27—N2—C21—C22	−100.24 (19)
S22—Cd1—S21—C27	9.02 (5)	C28—N2—C21—C22	85.9 (2)
S12—Cd1—S21—C27	−75.59 (6)	C26—C21—C22—C23	−0.5 (3)
S11—Cd1—S21—C27	−127.39 (5)	N2—C21—C22—C23	−177.74 (15)
N3—Cd1—S22—C27	−104.87 (6)	C21—C22—C23—C24	1.6 (3)
S12—Cd1—S22—C27	143.59 (5)	C22—C23—C24—C25	−1.8 (3)
S11—Cd1—S22—C27	66.81 (6)	C23—C24—C25—C26	0.9 (3)
S21—Cd1—S22—C27	−8.91 (5)	C22—C21—C26—C25	−0.3 (3)
S22—Cd1—N3—C33	−165.44 (14)	N2—C21—C26—C25	176.91 (15)
S12—Cd1—N3—C33	−51.65 (16)	C24—C25—C26—C21	0.2 (3)
S11—Cd1—N3—C33	21.00 (16)	C21—N2—C27—S21	−178.05 (12)
S21—Cd1—N3—C33	122.78 (15)	C28—N2—C27—S21	−4.7 (2)
S22—Cd1—N3—C31	21.83 (15)	C21—N2—C27—S22	1.6 (2)
S12—Cd1—N3—C31	135.62 (13)	C28—N2—C27—S22	174.93 (14)
S11—Cd1—N3—C31	−151.73 (13)	Cd1—S21—C27—N2	165.05 (14)
S21—Cd1—N3—C31	−49.95 (14)	Cd1—S21—C27—S22	−14.54 (8)
C17—N1—C11—C12	92.93 (19)	Cd1—S22—C27—N2	−164.30 (13)
C18—N1—C11—C12	−90.55 (18)	Cd1—S22—C27—S21	15.31 (9)
C17—N1—C11—C16	−88.54 (19)	C27—N2—C28—C29	106.4 (2)
C18—N1—C11—C16	87.97 (19)	C21—N2—C28—C29	−79.9 (2)
C16—C11—C12—C13	1.2 (3)	C33—N3—C31—C32	0.5 (2)
N1—C11—C12—C13	179.69 (15)	Cd1—N3—C31—C32	174.66 (11)
C11—C12—C13—C14	−0.8 (3)	C33—O3—C32—C31	1.35 (19)
C12—C13—C14—C15	0.0 (3)	N3—C31—C32—O3	−1.1 (2)
C13—C14—C15—C16	0.4 (3)	C31—N3—C33—O3	0.4 (2)
C14—C15—C16—C11	0.0 (3)	Cd1—N3—C33—O3	−173.07 (11)
C12—C11—C16—C15	−0.8 (2)	C31—N3—C33—C34	−178.45 (18)
N1—C11—C16—C15	−179.31 (14)	Cd1—N3—C33—C34	8.1 (3)
C11—N1—C17—S12	−1.5 (2)	C32—O3—C33—N3	−1.2 (2)
C18—N1—C17—S12	−177.80 (12)	C32—O3—C33—C34	177.82 (15)
C11—N1—C17—S11	178.59 (11)	N3—C33—C34—C35	106.5 (2)
C18—N1—C17—S11	2.3 (2)	O3—C33—C34—C35	−72.4 (2)

### Hydrogen-bond geometry (Å, º)

Cg1 and Cg2 are the centroids of the C11–C16 and C21–C26 rings, respectively.

**Table:**

*D*—H···*A*	*D*—H	H···*A*	*D*···*A*	*D*—H···*A*
C26—H26···*Cg*1^i^	0.95	2.61	3.558 (2)	177
C32—H32*A*···*Cg*1^ii^	0.99	2.72	3.511 (2)	137
C13—H13···*Cg*2^iii^	0.95	2.61	3.510 (2)	157

Symmetry codes: (i) −*x*+1, −*y*, −*z*+1; (ii) −*x*+1, −*y*+1, −*z*+1; (iii) *x*+1, *y*, *z*−1.
